# P02.146. Mindfulness based stress reduction in adults with mild cognitive impairment: a pilot study using fMRI

**DOI:** 10.1186/1472-6882-12-S1-P202

**Published:** 2012-06-12

**Authors:** R Wells, G Yeh, C Kerr, J Wolkin, R Davis, Y Tan, R Wall, J Walsh, T Kaptchuk, D Press, R Phillips, J Kong

**Affiliations:** 1 Brigham and Women's Hospital/Harvard Medical School, Brookline, USA; 2 Beth Israel Deaconess Medical Center/Harvard Medical School, Boston, USA; 3 Brown University, Providence, USA; 4 NYU Langone Medical Center, New York City, USA; 5 Massachusetts General Hospital/Harvard Medical School, Boston, USA; 6 Commonwealth Care Alliance, Boston, USA

## Purpose

Fifty percent of adults with mild cognitive impairment (MCI) develop Alzheimer’s disease (AD) within 5 years. Preliminary data suggest that mindfulness-based stress reduction (MBSR) increases gray matter density of the hippocampus, which atrophies in AD. We studied the safety, feasibility, and impact of MBSR on brain function, memory, and quality of life (QOL) among adults with MCI.

## Methods

We randomized 14 MCI patients (2:1) to 8 weeks of standardized MBSR (n=9) or wait-list control (n=5). Brain activity with fMRI resting state, neuropsychological and QOL measures were assessed at baseline and 8 weeks; the latter two were also assessed at 6 months.

## Results

The mean (SD) age was 74 (7); baseline Mini-Mental State Exam score was 27.2 (1.5); class attendance was 88%; and home practice was 26.1 minutes/day (19.6). No adverse events were reported. At 8 weeks, episodic memory was not improved in adults randomized to MBSR compared to the control (median change [Q1, Q3] from baseline, Rey Auditory Verbal Learning Test total recall -2.5 [-5.5, 0] vs. +1 [-1, 4], p=.24). Non-significant trends that suggested improvement with MBSR were detected for change from baseline for MBSR vs. control for AD Assessment Scale-cognitive subscale (-0.5, [-4, 0.5] vs. 0 [-1, 2], p=0.46); Resilience Scale (+7 [2, 21] vs. -2 [-9, 0], p=0.18); QOL-AD (+2 [-1, 3] vs. 0 [-1, 0], p=0.25); Perceived Stress Scale (-1 [-6,0] vs. 0 [-4, 2], p=.46). Compared with the control group, the regional homogeneity of the left hippocampus and putamen/inferior frontal gyrus were significantly enhanced in the MBSR group. Data for 6 months are not yet available.

## Conclusion

MBSR was associated with changes in spontaneous brain activity in the hippocampus and putamen/inferior frontal gyrus in adults with MCI. A randomized controlled trial in MCI evaluating effects of MBSR on neuropsychological, behavioral, and neuroimaging measures is feasible and safe.

